# Positive events in psychotherapy: What do adolescents and young adults say is helpful?

**DOI:** 10.12688/f1000research.152349.2

**Published:** 2025-03-17

**Authors:** Luisa Cassera, Alessia Calabrò, Aschieri Filippo

**Affiliations:** 1 Psychology, Universita Cattolica del Sacro Cuore, Milan, Lombardy, 20123, Italy

**Keywords:** Psychotherapy, helpful events, adolescents, young adults, patient perspective

## Abstract

**Background:**

The significant challenge in adolescent and young adult therapy lies in the fact that research in this field does not provide many clinical indications. This study addresses domains contributing to the establishment and sustenance of engagement, satisfaction, and progress among adolescents and young adults in their treatment by delving into the impact of events that, from the perspective of clients, have positively influenced their therapeutic process.

**Methods:**

Employing a qualitative research approach, we conducted semi-structured interviews with 13 adolescents and young adults undergoing therapy, recounting positive and constructive events during their treatment.

**Results:**

A structured thematic analysis revealed six primary domains of positive events: events linked to the management of the setting, events shaping the definition of therapy goals, occurrences tied to the therapist’s active role, collaborative events between therapists and clients, and events associated with both clients’ external and internal worlds.

**Conclusions:**

The findings suggest that working with adolescents shares similarities with psychotherapy involving adult clients but demands specific attention to adolescents and young adults’ families, surrounding environment, and rapidly changing needs.

Adolescence, derived from the Latin “adolescentia,” indicates the transition period from childhood to adulthood. It is marked by significant physical, cognitive, emotional, and social changes. The conclusion of adolescence, typically around age 19, is defined by the acquisition of developmental tasks that help individuals build their identity and prepare for adulthood. These tasks, as described by Havighurst (1948), contribute to happiness and success when completed but lead to difficulties when unmet. In recent years, a new life phase, post-adolescence or young adulthood, has emerged in Europe, reflecting prolonged dependence on the family of origin and delayed identity formation. This is paralleled by the American concept of Emerging Adulthood, where young people explore various life directions ( Arnett, 2002). Young adulthood is a crucial period, showing both differences and similarities with adolescence.

These years between 18 and 26 represent a period of psychological development, where young adults bridge the differences between adolescence and adulthood, influenced by new roles, responsibilities, and social context changes ( Cauffman et al., 2010). Compared to adolescents, young adults take more time to consider difficult decisions, are less influenced by rewards, are more sensitive to potential costs, and have better impulse control.

Despite these peculiarities, adolescents and young adults share several similarities, which is why they are often grouped together as a single population of interest. Both groups, in fact, go through a transitional period characterized by internal and external movements, as well as a continuous search for separation and individuation ( Havighurst, 1948). Socially, the transition to adulthood is becoming more unpredictable and is increasingly shaped by individual choices rather than social structures (Steinberg, 2013; Settersten & Ray, 2010). Although young adults engage in less risky behavior and are healthier than adolescents, they live more independently from parents and have less supervision, which can increase vulnerability. These features may extend the period of potential harm often associated with adolescence ( Harris et al., 2006; Neinstein, 2013; Schulenberg & Maggs, 2002). Also, although adolescence and young adulthood are distinct phases, both involve continuous cognitive and motivational changes in the brain that can affect the ability to understand and negotiate with others. This makes the transitional phase a period of uncertainty and opportunity, where social and institutional support is crucial to facilitate a path toward a more stable future (Arnett, 2004; Furstenberg, 2010; Roisman et al., 2004).

Another important aspect that unites the two developmental stages described is the therapeutic alliance, which in psychotherapy with adolescents and young adults assumes a collaborative dimension. It should be considered that the independence and self-determination typical of these developmental stages can serve as significant normative-evolutionary obstacles that may complicate the achievement of an agreement between client and therapist ( Karver et al., 2018; Meeks & Bernet, 2001). Moreover, it seems beneficial to involve caregivers, who are often responsible for bringing them to therapy and/or covering its financial cost ( Cirasola et al., 2021). This implies that the construction of long-term therapeutic goals can be complex, especially if the young person has little self-awareness of their problems or cognitive difficulties, which are also influenced by brain changes occurring during adolescence and young adulthood ( Bonnie et al., 2015).

There is substantial evidence regarding the effectiveness of psychotherapy with children and adolescents ( Goodman, Calderón, & Midgley, 2022), more so when the therapists follow evidence-based psychotherapies (EBPs) ( Weisz et al., 2013) such as Multisystemic Therapy (e.g., Timmons-Mitchell et al., 2006), Cognitive-Behavioral Therapy (CBT; e.g., Stallard, 2022), Interpersonal Therapy (IPT; e.g., Gunlicks-Stoessel et al., 2010), Psychodynamic Therapy (PDT; e.g., Midgley et al., 2021), and Mentalization-Based Treatment (MBT; e.g., Rossouw & Fonagy, 2012).

Goodman and Halfon (2021) found two successful treatment paths for children: one focused on mentalization, which improved emotional regulation, and another centered on providing support, which helped, for example, contain oppositional behaviors. Mentalization, by fostering trust and collaboration, enables patients to learn from new experiences and adjust their understanding of social relationships. Providing support, offering encouragement or approval to patients, helps them cope with stress, challenges, or emotional difficulties. These processes might be more important than specific therapist knowledge shared during therapy.

The investigation carried out by Goodman, Calderón and Midgley (2022) involving thirty-one expert clinicians from diverse therapeutic backgrounds indicated the presence of two effective processes in adolescent therapy across four distinct presentations of widely adopted therapy models (Psychodynamic Therapy- PDT; Mentalization-Based Treatment- MBT; Cognitive-Behavioral Therapy- CBT; Interpersonal Psychotherapy- IPT): the promotion of mentalization and the provision of support. Utilising the Adolescent Psychotherapy Q-Set (APQ), the researchers found PDT and MBT seem to involve the change process by promoting mentalization, while CBT and IPT seem to change process by providing support.

Cirasola and Midgley (2022), in their critical review of alliance with younger clients, emphasise its role as a crucial mechanism of change in their psychotherapy. Over the last few decades, researchers have made efforts to better understand the temporal precedence of the alliance and symptom change through sophisticated designs and analyses ( Falkenström & Larsson, 2017; Zilcha-Mano et al., 2014). In youth psychotherapy, some studies have identified a significant association between early alliance and later symptom severity, even when controlling for initial severity ( Chiu et al., 2009; Labouliere et al., 2017; Marker et al., 2013). More recently, Cirasola et al. (2021), using data from the IMPACT study, a randomised controlled trial comparing cognitive-behavioral therapy, short-term psychoanalytic psychotherapy, and a brief psychosocial intervention in the treatment of adolescent depression, controlling for pretreatment symptom severity and prior symptom change, found that average alliance ratings by adolescents (N = 224) and therapists (N = 139) early in therapy had a weak but significant association with subsequent symptom change.

Alliance with younger clients is particularly relevant also because adolescents’ motivation to participate and collaborate in treatment may differ from that of adults. The systematic review by Gulliver, Griffiths and Christensen (2010), covering both qualitative and quantitative literature on perceived barriers and facilitators to help-seeking for mental health problems in adolescents and young adults, highlighted that young individuals are often directed to treatment by others (such as parents, family, or teachers) and rarely seek therapy independently. Additionally, adolescents and young adults may lack motivation to seek therapeutic help because attending mental health services conflicts with their developmental needs, especially regarding peer acceptance, autonomy from parents, and their high dependence on parents to solve problems. So, while working with adolescents requires respecting their individuality, agency, and confidentiality as conditions for a robust alliance ( Gulliver et al., 2010; Wilmots et al., 2020), caregivers are often involved in their children’s therapy in various ways: they are frequently the reference source that may contribute to the initial assessment or some sessions, and even when not directly involved in their children’s treatment, they are often responsible for bringing them to therapy and/or its financial cost ( Cirasola et al., 2021). Additionally, it is essential to remember that, in the case of a minor, the consent of both parents or legal guardians is a crucial aspect for undertaking any form of treatment ( APA, 2002).

Consequently, therapists need to build an alliance not only with the direct stakeholders (adolescent or young adult) but also negotiate an alliance with their caregivers, often defining different goals as these may diverge between the young person and the caregivers. Working with parents is crucial to strengthen the youth’s alliance with the therapist. The ability to provide parents with psychoeducation, respectful challenges, empathy, and support at a deeper level than currently possible is likely to be beneficial, even though it might require more clinical hours dedicated to the caregiver/s. Clinical time can be saved by engaging in preventive work, shorter work, and potentially ensuring safer and more lasting outcomes with the adolescent ( Marks, 2020).

Without mutual commitment, parents/guardians can be seen as obstacles to their children’s treatment, and therapists working with adolescents may perceive them as intrusive or disengaged from the adolescent’s therapy. A sense of demoralisation about the prospects of fruitful engagement may become the professional response to parents who appear angry, inflexible, enmeshed, jealous, or avoidant ( Marks, 2020).

The significant challenge in adolescent and young adult therapy lies in the fact that research in this field does not provide many clinical indications, especially when working with adolescents from non-Western cultures in which adolescence is not defined in the same terms of individuation/separation as in Eurocentric societies ( Cirasola & Midgley, 2022). To fill this gap, qualitative research methods could provide insights about adolescents’ experiences of their treatment. The research on significant events in psychotherapy (any reaction, intervention, or response that resonates for the patient, which marks a turning point in the therapeutic relationship, Elliott, 1985) allows us to investigate in-depth meanings attributed by patients to their experiences in therapy.

A recent study conducted by Calabrò, Cassera and Aschieri (2024) examined the impact of events that hinder the therapeutic alliance in psychotherapy with adolescents. Adolescents remembered as particularly problematic events connected to therapists’ misuse of diagnosis, lack of responsiveness, inappropriate focus of sessions, abstractness, poor management of the differences in opinions between patient and therapist, non-response to their need for protection and patient blaming. Also, adolescents experienced problems in the management of the therapy setting, such as when they perceived session discontinuity, broken confidentiality and unclear boundaries between therapist’s private/work life. Participants to the study also reported as problematic their lack of internal motivation and internalised stigma about therapy, the interferences from their environment with regards to attending or interrupting therapy and their concerns for its economic sustainability. Facing these difficulties let participants feel a range of negative emotions, and painful experiences such as rejection, confusion and overwhelm. Despite the distress for the negative events they experienced, the discriminant element that emerged has been the way the therapeutic dyad was able to identify and repair the rupture in the relationship or not. In conclusion, all 14 participants considered psychotherapy potentially beneficial. Participants who opted to seek another therapist used the negative events encountered in their previous treatment as a guide for what they did not want to experience in the next one. Others left therapy with ambivalence, acknowledging both the negative aspects of treatment and some positive aspects that made them open to the possibility of starting a new treatment with a different therapist in the future.

Building on the findings of this research, the present study seeks to identify positive events categorised as helpful within the therapeutic setting. This endeavour aims to offer a deeper insight into patients’ psychotherapy experiences. In a future perspective, the gathered information will empower therapists to better understand their patients’ perspectives and adeptly manage constructive events occurring within the realm of psychotherapy.

## Objectives

This study aims to investigate the positive events that have facilitated the engagement and satisfaction for treatment with adolescents and young adults. Specifically, the goal is to deepen the patients’ perspective regarding how specific episodes in therapy were seen as helpful and positive, and which variables could contribute to promote clients’ appreciation of their therapy.

## Method

The research follows the significant events paradigm ( Arnkoff et al., 1993; Elliott et al., 2001) and the narrative-hermeneutic approach ( Bruner, 1986), focusing on the subjective experiences of adolescents and young adults with regard to significant events in psychotherapy. In this study, the interviews are analyzed to extract the key patterns of meaning. Thematic analysis ( Braun & Clarke, 2006) is employed to build a hierarchical system of categories based on the text. In this approach, the researchers do not begin with a pre-defined classification of events; instead, they start with the clients’ narratives to develop bottom-up an empirically grounded classification of events. As Bryman (2008) sustain, the amount of data collected in qualitative research is not fixed or calculable, but continues until saturation is reached. That is, study data were collected until emerging concept shave been explored and additional data were not producing fresh insights.

In this regard, the study followed the Consolidated criteria for reporting qualitative research (COREQ) ( Tong, Sainsbury & Craig, 2007). In the following sections we report in square brackets the corresponding number of each information to the list of criteria.

### Participants

**Recruitment, inclusion and exclusion criteria.** We recruited adolescents and young adults between the ages of 14 and 25. The inclusion criteria included at least four months of psychotherapy to allow for the development of a significant therapeutic relationship with the therapist ( Hilsenroth, Peters & Ackerman, 2004). Also, to avoid the bias associated with the recollection of distant events, participants had to be still actively involved in their therapy at the time of interview. We required all candidates to have experienced at least one positive and helpful event during their treatment. No specific exclusion criteria were set.

In qualitative research involving similar participants (in our case, adolescents) and a specific focus (in our case, positive events in their therapy) literature suggests a range of 9 to 17 interviews to ensure data saturation ( Hennink & Kaiser, 2022). Following the approach by Young and Casey (2019), saturation was determined by analysing the frequency of specific codes: transcripts were reviewed in sequence, tallying the appearance of new codes in each interview until no further codes emerged. New participants were interviewed until no further thematic categories emerged. In our research, we collectively agreed that thematic saturation was obtained after completing 13 interviews [12, 23].

**Sample characteristics.** The participants included in the study are native Caucasic Italian-speaking adolescents and young adults residing in Lombardy, Veneto, and Emilia Romagna, who were undergoing psychotherapy at the time of recruitment. The study included 13 adolescents (8 females) between 14 and 25 years old (M = 21.8). With regard to the region of origin of the sample, 4 interviewed were from Lombardy, 7 from Veneto and 2 from Emilia Romagna [14].

### Procedure

Convenience sampling was used [10]. An information letter about the study was spread through the collaboration of a network of psychotherapists working in different contexts (communities of minors, private practices, Public Mental Health Services) present throughout the North Italian territory. The information about the study was either delivered personally or posted by the colleagues at their respective workplaces to reach potential participants. Potential participants provided their availability to participate in this study to their therapists, which shared their contact information to the second author (L.C.). Prior to each interview, the Informed written consent was collected from each potential participant (or from their legal guardians, if clients were younger than 18 years old) and L.C. addressed and discussed potential participants’ questions and comments about their participation in the study. Due to the sampling strategy, it is impossible to determine how many potential participants were informed about the study but declined to take part [13].

Data was collected between May and July 2023. The average duration of each interview was 25 minutes (the shortest lasted 13 minutes, while the longest lasted 37 minutes) [21]. All interviews were conducted remotely using the online platform of Microsoft Teams [11]. Interviews were audio and video recorded and transcribed [19]. Two interviewers (a self-identifying male Ph.D. serving as researcher in a private university and one self-identifying female student of a Master program in clinical psychology [1, 2, 3, 4]) took notes of relevant topics for the eventual coding during the interviews [20]. Only L.C. and F.A. were present to all interviews [15]. The study conforms to the Declaration of Helsinki on the Ethical Principles involving Human Participants, and research protocol was approved by the IRB of Catholic University of the Sacred Heart, [approval number #51-23; 25.05.2023]. One participant asked to check the quotes from the interview for accuracy before confirming their use in the text of the manuscript [23].

### Instruments

The instrument used in this study was a semi-structured interview based on a review of the scientific literature on the positive and enhancing events of therapy. Areas of interest and questions were constructed on the basis of the paradigm of significant events introduced by Robert Elliott (1985). They were also constructed on the basis of the theoretical-methodological framework of the ENACT self-report questionnaire ( Kohrt et al., 2015) for which consent has been obtained from the corresponding author. The interview included questions such as: “During your psychotherapy experience, have you felt that you’ve encountered any events that you deemed significant/constructive for your journey?” and “Why do you consider this experience significant?” and allowed follow up questions to deepen the description and understanding of pertinent themes [17]. No repeat interviews were conducted since both interviewers were experienced in conducting this type of research, and had previously conducted other similar interviews (see, Calabrò, Cassera & Aschieri, 2024) [5, 18].

### Data analysis

The coding process of the interviews followed Braun and Clarke (2006), described in detail elsewhere ( Aschieri et al., 2021, 2024) [24, 26, 28], moving from the development of a first set of possible coding categories to a structured thematic map though recursive reading of the interviews and revision of the general map of the categories that were identified [25]. The final coding included two levels of analysis: main themes and themes. In line with Hennink and Kaiser (2022) and Aschieri et al. (2021), in this study we reported the frequency of subjects mentioning each theme. The results section also includes themes that are mentioned only one time.

## Results

Six typologies of helpful events emerged from the coding [31]: events connected with the *management of the therapeutic setting;* events related to the *definition of therapy goals,* events that qualify the *role of the therapist; collaborative events between therapist and client;* events connected with *clients’ internal world;* events connected with *clients’ external world.* Each theme was composed of different sub-themes [32]. Tables 1- 6 summarise the structure of the categorization. The text below reports one excerpt from participants for each sub-theme. More examples for each sub-theme are given in the online Appendix A [30].

**Table 1.** Management of the Therapeutic Setting.

**MANAGEMENT OF THE THERAPEUTIC SETTING**	Definition of Privacy Boundaries
	Availability Between Sessions
	Flexibility of Session Frequency

**Table 2.** Definition of Therapy Goals.

**DEFINITION OF THERAPY GOALS**	Addressing Adolescent’s Priorities
	Redefining the Style and Purpose of Therapy

**Table 3.** Role of the Therapis.

**ROLE OF THE THERAPIST**	Active Engagement
	Construction of Epistemic Trust
	Self disclosure
	Authenticity of Personal Relationship
	Emotional Responsiveness
	Affinity with the Client

**Table 4.** Collaboration.

**COLLABORATION**	Collaborative Use of Test
	Collaborative meaning-making
	Personalization of the Therapist's Relational Approach to the Client
	Client-Therapist Reciprocity

**Table 5.** Client’s Internal World.

**CLIENT'S INTERNAL WORLD**	Understanding One's Internal World
	Positive Connotation
	Addressing Shame
	Emotional Regulation
	Exploring “Uncomfortable” Issues
	Promoting Self- Acceptance

**Table 6.** Client’s External World.

**CLIENT'S EXTERNAL WORLD**	Work Directly with Parents and Significant Others
	Changing the Representation of the Relational Environment
	Suggesting Alternative Strategies for Coping with Adaptation Difficulties in the World
	Identifying and Involving Additional Attachment Figures

### Management of the therapeutic setting

The first theme includes events that qualify how the therapist organised the setting. We included in this category all examples of setting management that, from the clients’ perspective, contributed to positively influence the course of treatment and the construction of the therapeutic alliance. In this regard, three sub-themes emerged: definition of privacy boundaries; therapists’ flexibility in adjusting the frequency of the sessions depending on adolescents’ needs, and therapists’ availability between sessions ( Table 1).

**Definition of Privacy Boundaries *.*** For thirteen adolescents, establishing the confidentiality boundaries within the therapeutic relationship was crucial. This allowed adolescents to perceive the differentiation between the therapeutic dyad and the external world, and to feel free - and safe at the same time - in expressing their own difficulties, as illustrated by the example below [29]:

*Cl. 10: “She (my therapist) didn’t request (referring to bringing my parents into the session) maybe because the issue wasn’t really within the family. Let’s say she saw it as a secondary thing, which we still investigated, but in reality, we understood that it wasn’t the central focus of my therapy.” […] Perhaps (if she had involved them), I wouldn’t have felt so open in expressing things, laying them out even if she knew them because … it wouldn’t have been that same intimate thing that has developed between me and my therapist. I didn’t see it as a relationship between me and someone doing their job but as something more intimate and profound. Because of the involvement, the empathy that was there, the fact that she made me feel good, there was something beyond the work that was intimate, personal, and involving someone else (my parents) might not have made it so.”*

**Availability Between Sessions.** A substantial number of adolescents ( *n* = 5) stressed the importance of the help therapists offered them between sessions. In particular, adolescents appreciated the therapists’ willingness to offer psychological support, during crises, remotely - usually via a phone call - outside scheduled sessions. In all cases, patients read this availability as a demonstration of understanding and genuine caring beyond the therapy room:

***Cl. 1:** “One evening, it was quite late, and I was at home; I had hurt myself and was really in a bad state and had these bad thoughts. I didn’t know what to do, so I wrote a message to (the therapist’s name) who, despite it being almost midnight, practically called me immediately. […] He made me understand that I shouldn’t worry, that we would talk about it, and he had found a place for me the next day. And he practically made me talk and made me say everything I was thinking at the moment and then said, ‘Okay, now try to relax, go to bed, sleep, and come here tomorrow morning.’ And that was probably the only thing I needed.”*

**Flexibility of Session Frequency.** Consistent with the previous category, this sub-theme supports the importance of flexibility in psychotherapy with adolescents and young adults. In particular, five participants positively valued the therapists’ willingness to adjust the frequency of sessions according to the specific needs of their clients. Here is an example:

***Cl. 8:** “There are times when I increased the frequency of my sessions. Sometimes I have nothing, other times I have a lot to tell. I move a lot, so sometimes I decrease, other times I increase (the frequency of sessions), and there have been times when I said ‘Enough’ to therapy. In my opinion, when you use it with the right consistency, that is, when you deal with a theme, then you can address others but in a much more relaxed way. Now, since I have fewer issues than before, I maintain psychotherapy mostly to get to know myself.”*

### Definition of therapy goals

The second theme includes events related to the definition of the purposes of therapy. This theme was divided into two sub-themes: how therapists addressed adolescents’ priorities, and how therapists were able to redefine the style and the purpose of therapy depending on adolescents’ changing needs.

**Addressing Adolescents’ Priorities.** Eleven adolescents found the therapists’ promptness in assessing problems and identifying priority issues to be very effective. These patients appreciated the therapists’ ability to recognize the most urgent and pressing topics and to intervene on them. In some cases, breaking down the major problem - perceived as unsolvable - into smaller and more attainable parts allowed the adolescents to feel more capable and ready to address that problem.

***Cl. 2:** “I remembered something my therapist used to say, ‘When the house is on fire, first of all, you call the firefighters. After that, you think about discovering how it happened, why it happened, etc., etc., but not before … and then you’re okay, you’re calm, you’ve come out of the house, you’re serene.”*

**Redefining the Style and Purpose of Therapy.** Nine adolescents appreciated the therapist’s willingness to adapt the style of therapy according to their characteristics, needs, and most pressing issues to be addressed. In this regard, the therapist’s flexibility fostered patients’ perception of the therapeutic alliance as a unique and dynamic relationship, tailor-made for them and open to continuous renewal.

*Cl. 9: “"When I was younger, she (the therapist) guided me more in reasoning and explanations. Now, instead, we have a real trusting relationship, and I have matured. […] At the beginning, there were many strong issues that I brought up. For example, I couldn’t stay in class, couldn’t go out, and my anxiety limited me a lot. Once I went to the monumental cemetery, saw a grave, and collapsed to the ground. This is to make you understand how powerful my problem was, and she (referring to the therapist) encouraged me very concretely. Now, however, she doesn’t give me concrete advice anymore because she knows very well that I can figure it out on my own, and so the dialogue between us has become much more mature.” […] “I am the typical girl who presents problems in transitional phases, in change. For example, initially, I had a problem related to managing anxiety. Then, after finishing the academy to become an actress, I experienced burnout. I am always ‘Cyclical,’ as she defines me (referring to the therapist). At first, I didn’t know how to tell my parents that I wanted to be an actress, and that was a moment of change. After that, I had problems with panic attacks, I had blocks, couldn’t sleep … Then, I had an issue with the perception of my body.” […] “Once I overcame this block, it came back cyclically because of my work as an actress, where aesthetics are fundamental”.*

### Role of the therapist

The third theme pertains to situations in which therapists’ manner of “being in the room" and personal attributes facilitated the development of a meaningful relationship with adolescents. Specifically, this category encompasses events that, from the adolescents’ point of view, played an important role in creating the perception of an engaging, interactive, and forthcoming therapist, capable of consistently “keeping adolescents’ best interests in mind.” Adolescents also appreciated events in which therapists shared their own versions of adolescents’ struggles, were perceived as authentic, emotionally responsive and shared the same traits or interests.

**Active Engagement.** A substantial number of adolescents (n = 7) appreciated the active role of the therapist and emphasised its impact on satisfaction about therapy. In particular, this sub-theme refers to the therapist’s ability not only to listen and empathise with the young clients, but also to engage them by asking questions and showing interest in their narratives. In all cases, the therapist’s responsive approach reflexively fostered the patient’s curiosity, openness, and active involvement in therapy.

*Cl. 5: “My therapist is a very unique person, even in terms of creativity; he has a lot of imagination, and I really love that. Despite the notion that creativity decreases with age, he always has so much, and probably, this is the most beautiful thing about my therapist.” […] “Um, he dives into the experience, into the moment he is living, so I never can say no to him; not because I feel obligated, but because I am so curious.”*

**Construction of Epistemic Trust.** In the early sessions, 13 clients built trust in their therapist. They considered as significant therapist’s capacity to anticipate their emotions and behaviors. Consistent with this category, the concept of “Epistemic trust" ( Fisher et al., 2023) relates to the development of the client’s ability to regard knowledge conveyed by significant others, in this instance their therapist, as pertinent to themselves and applicable to other contexts ( Campbell et al., 2021). Let’s examine one example:

*Cl. 9: “It was nice because I thought, ‘Oh yes, this time she really understands me, gives me feedback.’ In short, to put it better, I could trust her because she ‘cradled’ me. I felt as if she were cradling me, ferrying me through what my situation was, also giving me advice on how to face my life.”*

**Self-Disclosure.** This category refers to the role of the therapist’s self-disclosure, known as crucial in facilitating the building of a genuine and authentic relationship with clients ( Audet & Everall, 2010). In five cases, the therapist’s self-disclosure allowed the adolescent to perceive him or her as a closer, credible, and trustworthy adult figure.

*Cl. 2: “(referring to the therapist) She gave me examples from her life and also from her work as a therapist to make me understand that very often I had too many preconceived ideas about where life would take me, the decisions I had made, and that they were more like negative forces, while in reality, I didn’t know. […] The fact of seeing the things we worked on in her as well […] that is, (the fact of ) giving me many examples of the idea of failure that I have and that maybe she also has. […] let’s say that certainly helped her credibility […]; because if she says, ‘Look, everything is fine, I know because I’ve experienced it, and it’s not like that. It doesn’t work that way.’ Life then becomes easier.” […] “Feeling in tune with the therapist at a certain point, maybe not regarding life goals but perhaps related to the same lifestyle, the same passions, was a beautiful thing. She was really excited and enthusiastic about the things I did because I felt that these were things she also enjoyed.*

**Authenticity of the Personal Relationship.** Ten adolescents describe therapeutic relationships as an ongoing construction, with their therapists, of an authentic and personal relationship characterised by mutual openness. Young clients have reported building a strong bond with their therapist through the intimacy and complicity that developed in the early sessions. The emphasis reported by adolescents and young adults is on the perception of the therapeutic setting as unique and special, constructed specifically for them in the unfolding of the therapeutic relationship. Here, one example:

*Cl. 11: “It was almost like talking to a friend when I told her things that had happened to me, and also how she reacted when I told her something funny, she laughed and played along, other times she advised me. Thanks to all this, complicity was created, so when I went to the sessions, I really wanted to tell her what had happened to me during the week. […] I think from the beginning, the way my therapist approached me made me perceive this complicity even though it matured over time.”*

**Emotional Responsiveness.** In six cases adolescents appreciated the therapist’s transparency in responding to some crucial moments of the therapy by showing their emotions to the clients. In particular, they perceived the therapist’s emotional response as an attempt to express closeness and humanity, in order to consolidate a more genuine and authentic relationship.

*Cl. 8: “Something I have always appreciated about her (my therapist) is that she empathises a lot. I mean, when I told her about this thing (For instance, I often smell my grandfather’s scent in my dreams), I could see her visibly with teary eyes; not that she ever cried excessively, but she had that emotion, which perhaps hits you even more than crying when you feel that here (points to the chest) everything is warm. You sense (referring to the warmth) in the other person when you share a common emotion. Well, I could say that the thing that she and I often share is common emotions.*

**Affinity with the Client.** Four adolescents found a number of commonalities with their therapist’s idiosyncratic characteristics in terms of personality traits, life experiences, passions, and interests. In all cases, the adolescent’s discovery of similarities with the therapist came as a pleasant surprise that fostered the perception of greater closeness between the two.

*Cl. 2: “In some way, another very positive thing, after ‘the bulk of the work,’ the most critical part had been overcome, that I noticed and liked a lot about my therapist, was the fact that in some way, as much as it was more like work she does on me, I felt like I had some affinity with her, you know!? So when I felt good, I felt that somehow she brought out the beautiful parts of me because she had them too. So the desire to travel, the desire to go on trips, particular adventures, things … I felt that she is like that because when she told me about her experiences (volunteering, travels, studies, etc.), I somehow felt connected; so it gave me the desire to do those things, right!? So a bit of affinity with the therapist is something that is positive, well.”*

### Collaboration

The fourth theme refers to the adolescents’ experience of collaboration with their therapists and has been found helpful in promoting the outcome of also very brief interventions ( Aschieri et al., 2023). Collaboration with adolescent clients appeared to be a cross-cutting process, which ranged from working together around the meaning of the psychological assessment data, to the co-construction of new meaning during the sessions, to the individualization of the relationship with every client, and to allowing (a certain amount of ) reciprocity of the care-taking process in the therapeutic dyad.

**Collaborative Use of Tests.** Five clients reported enjoying discussing collaboratively with their therapists about their psychological testing before the onset of the treatment. This fostered adolescents’ curiosity in themselves and became an occasion to enhance the disclosure of their subjective experiences.

*Cl. 11: “I have taken many tests, and I liked them because I didn’t feel the pressure to express myself without speaking directly in the moment. Still, I had the opportunity to work on certain things indirectly through these tests. […] Not having to speak directly, which I found challenging, was very helpful, especially when, after taking the test, we (my therapist and I) could discuss the results, etc. The tests also helped me better understand which areas to focus on and work on more.”*

**Collaborative Meaning-Making.** All adolescents reported that engaging in discussions with their therapist was beneficial to a better understanding of their issues. Dialogues with the therapist facilitated reflection on the relational context surrounding these issues and the analysis of their emotions and thoughts linked to them. This process enabled them to gain a clearer understanding of themselves.

*Cl. 12: “Speaking, going back in time (the therapist) would ask me, ‘Why did this thing happen here?’ ‘What happened before?’ ‘Why do you feel this way?’ And I could give myself answers that probably, before, I thought about, but I didn’t connect all the dots.” [ *…*] “Analyzing certain situations, sitting at the table. My psychotherapist told me about what was happening to me, ‘Let’s understand, not just see the problem, what’s around the problem? [ *…*] It made me think.”*

**Personalization of the Therapist’s Relational Approach to the Client.** Six adolescents perceived that therapists had tailored their relational approach to the unique characteristics of the patients resulting in a therapy process that is perceived as collaboratively defined by both parties. The perception of a customized work facilitated their active engagement in therapy and the building of a stronger relationship with their therapists. Here is an example:

*Cl. 10: “I am a very open person, so this thing (the therapist) really met me halfway, and I am very happy about that. I think maybe, if I had been more shy or less extroverted, it might have taken me more time, or I might not have opened up completely. This is because a person with a different personality (than mine) might have difficulties in connecting. Thanks to this encounter, I must say that a connection was immediately created.” […] “(referring to therapy) It’s a quite personal thing because I believe that each person is unique. So, I think, yes, the thread of the journey should have a similar starting point for everyone, but then, in reality, it diverges and unfolds based on the individual you have in front of you, on the problem they bring. In fact, I felt (the relationship with the therapist) was something personal; I don’t think (referring to the therapist) it’s the same with others. It’s a personal way of relating.”*

**Reciprocity Between Client and Therapist *.*** In five cases, the therapeutic relationship was strengthened by clients’ perception of a reciprocal engagement with their therapists. The adolescents emphasized their curiosity in their therapists’ lives, and, concurrently, the therapists’ willingness to share brief information about their daily lives. This mutual interest was viewed as helpful by the adolescents.

*Cl. 5: “We got along very well, me and him (my therapist), really well. Yes, yes, I found him, and he probably found me. […] I don’t even realize it anymore since there are no more (boundaries), there are no more formal barriers; there haven’t been to the point that, even before starting the session, I ask him ‘How are you?’. First of all, because I’m interested in knowing how he is, how his week went, in a short time, of course, and then the session starts in full. It’s a beautiful bond, I’m happy, really happy.”*

### Clients’ internal world

The fifth theme includes events centered on the adolescents’ emotions, thoughts, and self-representations. All participants recalled events in which they were helped having more understanding and compassion for themselves, less shame and a better emotional regulation. Adolescents experienced positive events in which their therapists accompanied them in exploring uncomfortable issues and themes, and validated their needs and desires towards more self-acceptance.

**Understanding of One’s Inner World.** For the majority of the adolescents interviewed ( *n* = 11) it was crucial to be supported by the therapist in analyzing their thoughts and emotions. This allowed them to “connect the dots" and mature a coherent and meaningful understanding of their inner world.

*Cl. 9: “This example is recent. I have had anxiety-related problems for a long time, and (my therapist) has practically suggested various ‘tricks,’ such as the ABC exercise, which is divided into different phases: situation, event, thought, etc … This helped me understand what the triggering thought (anxiety) was and what the (psychological) mechanisms underlying my behaviors and reactions are—not to avoid but to manage anxiety. (My therapist) helped me understand how, when you think about one thing, it can make you understand another, which, in turn, can be connected to another. So, thanks to these steps, you can understand why you are behaving in that particular way.”*

**Positive Connotation.** Positive connotation refers to the process whereby the therapist guides the client to re-read problematic and negatively judged behaviors in a different key, in order to construct a new self-narration. Specifically, the positive connotation allowed six adolescents to access a new perspective on psychopathology, as an attempt to adapt to intolerable conditions, rather than as a deficit.

*Cl. 13: “I have never had complete, blind trust in other people; especially during that time, I expected to be stabbed in the back by anyone. Having someone (referring to the therapist) who at least understands the situation, didn’t blame me for that, was immediately beneficial. […] From the second visit I had, this negative perception (of people) disappeared because (my therapist) showed me she appreciated the value of that feature of my personality. Despite understanding my weaknesses, he chose not to use them to his advantage. […] During the second visit, the doctor read me the data that came out of the Rorschach test, and some of them, such as patience, were above the limits, that is, they literally scored the maximum points. And (my therapist) instead of telling me they needed to be corrected, said that I had to be very brave to be so patient and at the same time be so guarded from other people”*

**Addressing Shame.** A consistent number of adolescents ( *n* = 8) talked about shame, recalling times when their therapists helped them overcome this emotion and its impact. In all cases, the therapists normalized shame, making sense of the distress caused by its symptoms or consequences, and making it understandable and accepted within the therapeutic relationship.

*Cl. 1: “I was helped to be and find the real me, and to normalize things. This, in my opinion, was so important because, more than anything, we live in a world that shames people with issues, such as depression and depressive disorders, and sees them as crazy or similar, which is a completely wrong perception" […] “(My therapist) didn’t make me feel the weight of the thoughts I had, like those about alcohol, suicide, or self-harm; he always somewhat normalized the issue, no matter how harsh and ugly it was" […] “It’s precisely for this reason that I am still here now because, probably, if I had found another psychologist who had said, ‘No, don’t do this, do that, go, I’ll send you to psychiatry, goodbye,’ I would have felt shattered" […] “So, when we find ourselves experiencing certain situations, we feel ourselves crazy and out of touch with the world, while he (the therapist) was good at making me understand that things were okay, it was normal after all, after so many things I had gone through; he managed to help me live through it with more serenity, and that helped me.”*

**Emotional Regulation.** In the majority of cases ( *n* = 9), adolescents reported that working on emotions was crucial. In this regard, the therapeutic relationship enabled them to verbalize -and legitimize -a wide range of emotions that included also those “negative emotions" that patients did not allow themselves to safely experience outside of therapy. In all cases, patients positively valued the help that therapists offered in terms of recognizing, accepting, and regulating the emotions which otherwise would have overwhelmed them and caused significant distress.

*Cl. 7: “For example, I used to think for a long time that anger, as an emotion in general, was a bad, unpleasant, and unjustified emotion […] and I classified it as a ‘Bad emotion, you shouldn’t feel it.’ Over time, I realized that all emotions are worth experiencing and have their reasons.” […] “So, I’m trying to learn how to express anger.”*

**Exploring “Uncomfortable" Issues.** Nine adolescents talked about the importance of addressing uncomfortable topics with the therapist. These were embarrassing or disturbing topics that were taboo outside of therapy. Among them, sexuality was one of the most recurrent. Here is an example:

*Cl. 8: “For example, (my therapist) would ask me, ‘What do you like, and how do you like to see yourself dressed? What made you discover a great pleasure for fashion?’ And I tried to answer those questions with her and put my answers into practice. For example, being a fairly feminist girl, at one point, (to the therapist) I even said ‘I masturbate,’ and she took it as an exercise, encouraging me.” […] “She also suggested that I go shopping alone, which was very difficult for me. I had panic attacks because I suffered from agoraphobia, which was really bothering me. From that moment on, I started to almost always go shopping alone, meaning it’s a moment I dedicate to myself.”*

**Promoting Self-Acceptance.** For a substantial number of participants ( *n* = 8), reflection on the inner world fostered not only better self-understanding but also greater self-acceptance. In this regard, the mirroring function of the therapists helped them integrate emotions, thoughts and parts of self that had hitherto been denied, repressed or expelled. This process enabled the adolescents to construct a self identity that was complex, multifaceted but also integrated and structured.

*Cl. 8: “For example, (my therapist) would ask me, ‘What do you like, and how do you like to see yourself dressed? What made you discover a great pleasure for fashion?’ And I tried to answer those questions with her and put my answers into practice.”*

### Clients’ external world

The sixth theme refers to events related with adolescents’ relationships outside the therapy. Four sub-themes emerged. In some cases, with the agreement of the adolescents, the therapist invited the parents or the significant others into sessions and worked directly with them. In other cases, the adolescents and therapists worked indirectly on these relationships, either by modifying for the representation of the relational environment or by suggesting alternative strategies for coping with others. Finally, when adolescents needed more support, therapists helped them to identify and involve additional attachment figures in their lives.

**Working Directly with Parents and Significant Others.** In four cases, adolescents benefited from the direct involvement of significant others in therapy. In particular, establishing contact with the patients’ parents was crucial. After ascertaining the adolescents’ consent, the therapists invited their parents into the session, involving them in a process sometimes parallel, sometimes joint with that of the young clients.

*Cl. 8: “I believe that she (my therapist) understood that when I started therapy, the major difficulty was related to the fact that I wanted to become an actress, but my parents didn’t accept it […] so she had to establish a dialogue with them too. Yes, she invited them (to therapy) but without me. I mean, there has never been a confrontation all together. It was only a confrontation with them to better understand who they were. Well, this thing is great; I really liked it when she made me understand that some situations, behaviors, etc., were not just my mistakes when she took my parents and said to them, ‘Come and talk.’”*

**Working Indirectly with Parents and Significant Others.** In other cases ( *n* = 11), adolescents and therapists worked indirectly on the patients’ significant relationships outside of therapy. This sub-theme includes clients who reported that they changed the representation of their relational environment, modifying their perception of other people and the way they related to them.

*Cl. 12: “Becoming aware that people cannot change but can improve. I have always tried to change people because they didn’t behave correctly with me, while she (referring to the therapist) once explained to me that I don’t have to change the person but try to make them think, to improve on certain things. Then there are those who want to do it and those who don’t. That’s a choice people make. Maybe I can take a step towards others by improving myself and automatically see a step from others towards me.”*

**Suggesting Alternative Strategies for Coping with Adaptation Difficulties in the World.** For a substantial number of participants ( *n* = 8), being able to apply the outcomes of the therapeutic work to daily life was essential. In this regard, adolescents appreciated the therapist’s ability to support them in dealing with problematic issues in daily life by suggesting alternative coping strategies and assigning them concrete tasks. This increased the perception of the therapy’s effectiveness.

*Cl. 8: “My mother is someone who wants a lot, controls a lot, and I am also someone who hyper-controls. Many times, she (my therapist) gave me specific tasks and even suggested that when I have a damn panic attack, I shouldn’t call my mother. From there, I noticed a significant and visible improvement. Sometimes she would say, ‘When this happens’ (referring to the panic attack), why don’t you call your dad?’ So, my therapist would say, ‘Involve your dad, talk to your dad,’ who, in fact, was a figure that I perceive as much quieter and with whom I clash much less, but with whom I struggled much more to be accepted on the artistic side. So, it was a concrete task for everyone (the whole family), even my sister who also suffers from panic attacks … “*

**Identifying and Involving Additional Attachment Figures.** The last sub-theme refers to the therapist’s role in building a social support network around the patient. In two cases, the therapist helped in the identification of additional professional help figures (e.g., psychiatrist, educator) who, alongside the parental couple, enabled the adolescents to feel more supported in the treatment process and in daily life in general.

*Cl. 3: “I understood what therapy is when the doctor also offered me another external support. At a certain point, he asked me if I felt the need to see someone, perhaps more often than him, who was an educator. […] We spent more hours together, talking about what I might say to the doctor in therapy and other things. Additionally, she physically helped me, or rather, supported me in my studies by giving me some methods to try or something like that.”*

## Discussion

Consistent with Burton and Thériault (2020) and Hilsenroth et al. (2004), most of the adolescents who participated in this study attributed the cause of meaningful and constructive events in therapy to specific characteristics and actions of the therapist. All participants (N = 13) reported that significant events occurred a few months after the beginning of therapy (3-4 sessions), aligning with literature indicating the initiation of therapeutic alliance construction during this period ( Burton & Thériault, 2020; Hilsenroth et al., 2004).

The six-themes derived from this study focus on several elements that our participants deemed relevant in their treatments. First, the emphasis on the management of therapeutic setting suggests that the therapists’ responsibility is to frame the “rules” of the sessions, stressing the importance of the definition of the “ *how*" rather than the “ *what*" (i.e., the content) ( Safran & Muran, 2003) particularly when working with this type of clients. A common feature among sub-themes emerging in this domain, such as *Definition of Privacy Boundaries, Availability Between Sessions and Flexibility of Session Frequency* was their change with time. The boundaries of privacy, the availability of therapists between scheduled sessions and the frequency of the sessions were flexibly renegotiated between adolescents and their therapists, allowing a better fit between the structure of the therapy and the changing needs of clients.

Also the theme *Definition of Therapy Goals* and sub-themes *Addressing Adolescent’s Priorities* and *Redefining the Style and Purpose of therapy* stress the positive appreciation for a patient-centered care, in which therapists follow adolescents’ agendas and flexibly adjust the goals and the process with which the treatment is delivered to adolescents’ changing requests and needs. Specifically, eleven out of thirteen adolescents emphasized the significance of therapists’ ability to address their priorities. This finding aligns with recent literature on assessing and accommodating therapy to adult patients’ preferences ( Cooper et al., 2023). Jacob et al.’ study (2022) found similar conclusions with adolescents. They underlined the importance of collaborative goal setting and agreement between young individuals and their therapists regarding personalized and meaningful outcomes on specific topics.

The theme *Role of The Therapist* stresses the importance, for adolescents clients, of some features that qualify their therapists’ behaviors and attitudes in treatment. The adolescents we interviewed appreciated events in which their therapists displayed their own personal characteristics and caring attitudes toward their clients in the therapeutic relationship ( Egozi, Tishby & Wiseman, 2021). What emerges from the analysis of the sub-themes ( *Active Engagement, Construction of Epistemic Trust, Self-disclosure, Authenticity of Personal Relationship, Emotional Responsiveness, Affinity with the Client*) is the relevance of the therapist’s self for adolescents. Being able to show care, interest, and responsiveness help adolescents develop trust in the importance of reaching their goals in therapy and attending carefully to the therapists’ views and advice. In the interviews we collected, affinity between clients’ and therapists’ personal features emerged at a later stage during the therapy, and it seems to be something that adolescents saw and built “on top” of their transformative experience, or at least after it started to unfold.

The *Collaboration* theme emerged in all interviews, and encompassed various activities, so that in our data it seemed to be a cross-cutting process that adolescents valued in many different circumstances, even when not explicitly mentioning it. In the sample of participants we interviewed, five adolescents completed with the same therapist a collaborative assessment prior to the onset of the actual treatment. Such assessment was explicitly considered by them as an integral component of their therapy, and was appreciated because it allowed adolescents to give their own versions of what the test scores mean for them, adding their individualised meaning to test data. The collaborative meaning-making, noticed by all participants, started in many cases from the discussion of the testing results in which therapists and adolescents tried to come up with joint understandings of the clients’ struggles observed through the lenses of assessment results. In other cases, collaborative meaning-making was conveyed by the use of open questions from the therapist, through which adolescents could make connections between previously unrelated aspects of their lives and experiences. Collaboration encompassed also the development of “ *ways of being in the room* “unique to each adolescent-therapist dyad, and such intimate and deep connection was accompanied in some cases by an authentic care for the person of the therapist by the clients.

The fifth theme ( *Client’s Internal World*) included processes that focused on the emotional, cognitive, narrative and representational aspects of adolescents’ functioning. The adolescents we interviewed appreciated being helped in developing greater self-understanding. The quality of the improved understanding about themselves seemed to have two specific features. First, it contrasted shame for their supposed inadequacies, either by normalising it or by judicious self-disclosures on the part of the therapist. Second, it framed symptoms, behaviors or problems that were previously deemed as “negative” by clients as smart adaptations to less-than-optimal environments provided them by their parents or social environment. Within this context of understanding, adolescents were able to appreciate the healing effect of sharing topics that were hard for them to address, regulate painful emotion with the support of the therapist, eventually accepting more of their experiences.

The *Client’s External World* theme underlined the importance of addressing directly and indirectly the context in which adolescents live their everyday life. Some adolescents we interviewed reported a benefit to the treatment from the real inclusion of their parents to some of the sessions. During such sessions, therapists helped adolescents and their parents to discuss problematic issues that were lingering in their relationships, and to attain better communication among family members. In some other cases, therapists helped adolescents to revise their view of their parents, or approach problems in their life differently, which led to a new way of dealing with issues outside the room of the therapy. Finally, adolescents appreciated the involvement and support from therapists when they felt that their relational network needed more structure and ressources.

### Limitations

It is important to consider some limitations of this study. Given the retrospective nature of this research, participants expressed experiences that occurred up to eight years before the interviews. The retrospective account of constructive and collaborative events might not be entirely faithful to what actually happened during the sessions. Instead, it could be a result of a reprocessing of events exposed to memory alterations and influenced by subsequent life experiences that over time contributed to giving a particular meaning to those events. Also, in this study we did not control for the overall satisfaction of clients for their treatments using valid measurement tools ( Di Malta et al., 2023), hence it is unclear if the perception of positive events is influenced by the general evaluation of the whole treatment.

The limited size and variability in the composition of the sample could be considered a potential threat to external validity, limiting the generalizability of results from the interviewed sample to the broader population of adolescent and young adult patients. Considering the small number of participants, potential gender differences were not investigated. Additionally, due to the absence of cultural diversity among the study participants, the conclusions and implications of our study should be restricted to Italian clients. The study did not account for potential internal distinctions within the sample related to psychotherapy, such as therapist orientation, location of sessions, or the reasons for therapy. These variables could be investigated in future research to examine their role in patients’ experiences, offering a more precise and accurate description of their lived experiences.

Finally, another limitation encountered concerns the duration of the interviews. The average duration of each interview was 25 minutes (the shortest lasted 13 minutes, while the longest lasted 37 minutes). The relatively short duration of the interviews may not have fully explored the significant events described by the participants. Therefore, replicating the present study with a deeper focus made possible by an increase in interview time could help better capture the core of the events and why they were considered significant by adolescents and young adults in their psychotherapeutic journey.

### Implications for research

Future studies could employ larger and more diverse samples, consider therapist characteristics and therapy settings, and incorporate more objective measures alongside qualitative narratives for a comprehensive analysis of therapeutic outcomes. Perhaps, most importantly, given the relevance of parents’ involvement in adolescents’ treatments, exploring their perspectives of what helps and what is hindering change in their children’s psychotherapy could be an interesting complement to the findings of this study.

## Conclusion

Therapy with adolescents can be a rewarding experience for therapists, but poses several challenges. How to keep in mind the family concerns for their children, foster alliance with the parents, and validate adolescents’ realization of the shortcomings of their environment? How to maintain the boundaries of privacy with adolescents when planning to do family sessions with their relatives? How to maintain a professional role with clients who highly value authenticity, openness and reciprocity in their treatment? How to be collaborative with adolescents, value their input in conjoint meaning making and also expand their views and self-understanding by adding information about their functioning?

The recent article by Calabrò, Cassera, and Aschieri (2024), addressing adolescents’ perceptions of hindering events in their therapies, illuminated several potential pitfalls for therapists when responding to these challenges. The current study indicated that therapists effectively navigated such obstacles, as reported by the participants, and helped to shed light on what adolescents valued in their therapy, offering an informative perspective on “what worked” in their treatment.

When comparing the main findings of Calabrò and colleagues with the current study, first it becomes evident that the management of certain relational variables plays a crucial role in guiding therapy towards a positive trajectory. These variables include: the therapist’s alignment with the adolescent’s objectives, the utilization of diagnosis to enhance self-awareness and foster epistemic trust in clients, the incorporation of therapy into adolescents’ daily experiences rather than engaging solely in abstract discussions, the facilitation of open dialogue and collaboration encompassing both areas of agreement and disagreement between adolescents and therapists, and the demonstration of authentic care and concern for adolescents’ well-being by therapists.

Second, the interventions that were deemed as most positive were those protecting adolescents from their shame, and those that provided a new, more compassionate and understanding perspective on themselves. One could wonder if the lack of these interventions might have been connected with the shame, stigma, lack of internal motivation lamented by participants in Calabrò et al. (2024).

Third, the management of the setting appears to hold significant importance. This includes remaining flexible to adolescents’ needs while ensuring session continuity, maintaining openness and a personalized approach while upholding clear boundaries between personal and professional lives, and involving parents in therapy while respecting confidentiality with adolescents. These tasks were identified by adolescents interviewed in both studies as the key focal points for therapists.

These seems to be common therapeutic factors in adolescents’ therapy and are present in different therapeutic approaches. As early as 1988, Fishman stated that “ *the most powerful therapeutic intervention at a social level for working with adolescents is family therapy*" (p.4) because the family is the “ *crucial point*” in every adolescent’s life. In systemic family therapy, the goal of the intervention is to reframe the negative views of parents for “ *dysfunctional behaviors*” of adolescents as symptoms of a larger set of needs in the family that need to be acknowledged and seen to promote the development of the system ( Aschieri, Fantini, & Bertando, 2013).

In the Trauma-Focused Cognitive Behavioral Therapy (TF-CBT) model, parents play a key role in the ongoing care of the child. In an interview with thirty youths aged 11 to 17, Dittmann and Jensen (2014) found that it is important for children to recognize their parents’ ability to handle hearing about their experiences, as it can help restore the parents’ role as a “protective shield” ( Pynoos, 1994). Although the youths valued confidentiality and predictability in what was shared during sessions, many found that sharing information was beneficial and appreciated.

In the 4-C model (competence, consent, confidentiality, and competing interests), it is important to discuss confidentiality with decision-making authorities, such as the child, parents, or guardian, before treatment begins ( Sori & Hecker, 2006; Sparta & Koocher, 2006). As Koocher (2008) notes, successful therapeutic alliances rely on mutual trust and privacy of sensitive content ( Harbour, 2004). It is essential to recognize the legal authority of parents or guardians and their interests in the child’s well-being, while aiming for a mutually acceptable understanding of confidentiality. Adolescents may prefer to discuss private matters, such as peer conflicts, school issues, smoking, alcohol, and sex, without their parents. Establishing clear boundaries around necessary secrets and when disclosures are necessary, such as threats of harm, helps clinicians negotiate acceptable limits with all parties at the beginning of treatment.

Another resource for therapists working with adolescents is the Therapeutic Assessment with families with adolescents’ model (TA-A; Tharinger et al., 2013). Despite being designed as a short-term consultation and presenting more research on its effectiveness with adult clients ( Durosini & Aschieri, 2021), Therapeutic Assessment offers various solutions to navigate in the complexities of working with adolescents and their families.

In Therapeutic Assessment (TA-A), the central focus lies in addressing both parents’ questions about their child and adolescents’ questions about themselves. Within this framework, privacy boundaries are determined by the questions posed by participants. Specifically, assessors initially agree with adolescents to disclose all pertinent information to parents to address their queries about the adolescents. Simultaneously, assessors request parents to respect complete privacy regarding information concerning adolescents’ inquiries about themselves. This agreement enables assessors to collaborate with adolescents in exploring answers to all questions through testing and interviews, and to jointly plan the inclusion and shaping of responses to parents’ inquiries. Collaborating with adolescents in this manner helps them feel that the assessment prioritises their concerns, while also allowing parents to perceive that the therapist/assessor is addressing their own objectives. Building on this foundation, collaboration during the assessment progresses to individualizing tests interpretation based on adolescents’ input and to link their testing outcomes to life challenges, both identified by parents and recognized by adolescents themselves. Therapeutic Assessment ( Finn, 2007; Fantini et al., 2022), rooted in intersubjective and humanistic psychology, encourages assessors to cultivate an open and supportive rapport with adolescents, embodying a balance between “ *compassion and firmness*” ( Finn, 2005) that aligns with adolescents’ need for support and guidance as well as their desire to gain insights into their psychological functioning. This model of authentic and collaborative involvement of adolescents and their families exemplifies a promising strategy for initiating and sustaining a productive therapeutic alliance, not only with adolescents but also with their families.

Given that the research has documented the association of the therapeutic alliance, also defined as the consensual or collaborative bond between clinician and client ( Finn & Tonsager, 1997; Shirk & Karver, 2003), with treatment retention, participation, and outcomes of therapy for young adolescents (e.g., Castro-Blanco & Karver, 2010; Shirk & Karver, 2003), it could be argued that the present study contributes to a better understanding of how clinicians can form a strong and constructive therapeutic alliance with their young clients.

Adolescents and young adults differ from adults both psychologically and socially, leading to the need for new frameworks like Emerging Adulthood ( Arnett, 2002) and Young Adulthood ( Confalonieri & Grazzani Gavazzi, 2002) to better understand their experiences. These developmental stages influence how therapeutic alliances form and evolve during therapy.

For adolescents, the therapeutic relationship is characterized by a need for trust, emotional closeness, and respect for their individuality, action, and confidentiality ( Cirasola et al., 2021; Gulliver et al., 2010). This is crucial for building a strong bond with the clinician, as adolescents may feel more engaged when they are deeply heard and understood. In addition, for both adolescents and young adults, therapy must consider their unique developmental stage, as emotional needs and autonomy differ from those of adults.

A key difference in youth psychotherapy is the involvement of caregivers, which is less prominent in adult therapy. The alliance with caregivers, whether through initial assessments or ongoing support, plays a central role in therapy. In this regard, several studies provide evidence that parental control plays an important role in the lives of adolescents ( Bean et al., 2006; Lamborn & Felbab, 2003; Hoeve et al., 2009; Kincaid et al., 2011; Inguglia et al., 2022). Therapists must balance the alliance with both the young client and their caregivers, who often have diverging goals and perspectives. Parental involvement can significantly impact treatment, reinforcing the therapeutic alliance and helping to navigate the delicate balance between fostering independence and providing necessary support ( Cirasola et al., 2021).

In conclusion, adolescence and young adulthood represent distinct developmental stages that influence the therapeutic process. Therapists must tailor their approaches to these stages, emphasizing trust, respect for autonomy, and the crucial role of caregivers in fostering a positive therapeutic outcome ( Cirasola & Montinari, 2012; Gulliver et al., 2010).

From a future perspective, the results of this study could help therapists working with adolescents and young adults to better understand their clients’ viewpoints and to adequately manage significant events that might serve as an important “bridge” to engage the adolescent throughout the course of psychotherapy. Regarding potential future research directions, it is possible to hypothesize that there may be specific differences within the sample according to the age of the client, particularly between adolescents and young adults. This could be explored in future research to identify those significant events that can improve clinical work with a focus on adolescence or young adulthood.

## Ethics and consent

The study conforms to the Declaration of Helsinki on the Ethical Principles involving Human Participants, and research protocol was approved by the IRB of Catholic University of the Sacred Heart, [approval number #51-23; 25.05.2023]. Prior to each interview, the Informed written consent was collected from each potential participant (or from their legal guardians, if clients were younger than 18 years old) and L.C. addressed and discussed potential participants’ questions and comments about their participation in the study.

## Data Availability

The files, including the complete Italian verbatim transcripts of all the interviews, were uploaded to Figshare open access repository. Figshare: Transcripts of the interviews. Dataset. https://doi.org/10.6084/m9.figshare.26067931. ( Aschieri, 2024). Data are available under the terms of the Creative Commons Attribution 4.0 International license (CC-BY 4.0). The files, the semi-structured interview guide, were uploaded to Figshare open access repository. Figshare: Transcripts of the interviews. Dataset. https://doi.org/10.6084/m9.figshare.26067931. ( Aschieri, 2024). All extended data were uploaded to the Figshare open access repository. Figshare: Transcripts of the interviews. Dataset. https://doi.org/10.6084/m9.figshare.26067931. ( Aschieri, 2024). They include the English version of: “Appendix A," which consists of all categories and their related verbatim; the individual tables related to the specific categories; participant information sheets; semi-structured interview guideline and consent form (adult and child version).
