# Supplementary material for: Positive events in psychotherapy: What do adolescents and young adults say is helpful?
Source: F1000Res. 2025 Mar 17;13:1025. Originally published 2024 Sep 9. [Version 2] doi: 10.12688/f1000research.152349.2 (PMC11933785; doi:10.12688/f1000research.152349.2)
Supplement: Peer review history

# Reviewer report: Reviewer response for version 2

**DOI:** 10.5256/f1000research.179166.r371350

**Published:** 2025-03-24

**Authors:** Anna Sidis

**Affiliations:** 1 School of Psychology, University of Wollongong, New South Wales, Australia

This authors changes have satisfied any previous concerns, this paper can now be approved.

Is the work clearly and accurately presented and does it cite the current literature?

Yes

If applicable, is the statistical analysis and its interpretation appropriate?

Not applicable

Are all the source data underlying the results available to ensure full reproducibility?

No

Is the study design appropriate and is the work technically sound?

Partly

Are the conclusions drawn adequately supported by the results?

Yes

Are sufficient details of methods and analysis provided to allow replication by others?

Yes

Reviewer Expertise:

Youth Mental Health, qualitative research, lived experience research, psychotherapy process research.

I confirm that I have read this submission and believe that I have an appropriate level of expertise to confirm that it is of an acceptable scientific standard.

# Reviewer report: Reviewer response for version 2

**DOI:** 10.5256/f1000research.179166.r371351

**Published:** 2025-03-18

**Authors:** Cristiano Inguglia

**Affiliations:** 1 University of Palermo, Palermo, Italy

Thank you for carefully considering all comments; I believe you have answered my questions fully.

Is the work clearly and accurately presented and does it cite the current literature?

Partly

If applicable, is the statistical analysis and its interpretation appropriate?

Not applicable

Are all the source data underlying the results available to ensure full reproducibility?

No source data required

Is the study design appropriate and is the work technically sound?

Yes

Are the conclusions drawn adequately supported by the results?

Yes

Are sufficient details of methods and analysis provided to allow replication by others?

Yes

Reviewer Expertise:

My area of research is psychological adaptation of adolescents and emerging adults with particular attention to child-parent relationships

I confirm that I have read this submission and believe that I have an appropriate level of expertise to confirm that it is of an acceptable scientific standard.

# Reviewer report: Reviewer response for version 1

**DOI:** 10.5256/f1000research.167098.r364696

**Published:** 2025-02-21

**Authors:** Cristiano Inguglia

**Affiliations:** 1 University of Palermo, Palermo, Italy

I am very grateful for the opportunity to review this manuscript that addresses several domains that may improve the quality of psychotherapy with adolescents and young adults. I appreciated the qualitative approach and I guess that the findings of this paper can contribute to promote authentic and collaborative involvement of adolescents and young adults to the psychological treatment. I have only some minor comments that can be addressed through a minor revision. In particular, I suggest:

- To avoid one sentence paragraphs throughout the manuscript
- On page 3, when authors state that “….by promoting mentalization, while CBT and IPT seem to change process by providing support” it would be better to explain better how the change is promoted in these two groups of therapy models.
- Given that the paper focuses on both adolescents and emerging adults, it would be better to add in the theoretical background some considerations about the specific developmental period in which adolescents and young adults live, highlighting the differences between these two periods and the shared psychological needs. For instance, there is a huge literature on the differences between adolescence and emerging adulthood that can be useful for this purpose.
- The same considerations about the different needs of adolescents and young adults should be considered in the discussion and the conclusion also considering the bug age range of participants. I guess that could occur some differences in the therapeutic relationship according to the age of the client (adolescent vs. young adult).  

Is the work clearly and accurately presented and does it cite the current literature?

Partly

If applicable, is the statistical analysis and its interpretation appropriate?

Not applicable

Are all the source data underlying the results available to ensure full reproducibility?

No source data required

Is the study design appropriate and is the work technically sound?

Yes

Are the conclusions drawn adequately supported by the results?

Yes

Are sufficient details of methods and analysis provided to allow replication by others?

Yes

Reviewer Expertise:

My area of research is psychological adaptation of adolescents and emerging adults with particular attention to child-parent relationships

I confirm that I have read this submission and believe that I have an appropriate level of expertise to confirm that it is of an acceptable scientific standard, however I have significant reservations, as outlined above.

# Response

**Published:** 2025-03-09

**Authors:** Luisa Cassera

**Affiliations:** Catholic University of Sacer Heart, Italy

*Dear Reviewer,*

*Thank you for the valuable feedback. We are pleased that you appreciated the qualitative approach used, and we thank you for your collaboration in making this article clearer and more accessible to colleagues in the field.*

*Below, you will find the corrections made:*

1. *We apologize for the use of short sentences. The intention was to simplify and minimize the information conveyed to the most salient points of the research in order to avoid being overly wordy.  *
2. *Regarding arguments of mentalization and providing support, we have described in more detail how these were considered cross-cutting variables and why, according to the authors, they promote change.  *
3. *We have introduced in the theoretical background some considerations about the specific developmental period in which adolescents and young adults live, in order to highlight the differences between these two periods and the shared psychological needs.  *
4. *Connected to the third point and in line with the suggestion from the first reviewer, we have expanded the argument, explaining what new findings the study brings, how it could change clinical practice as a result, and finally, we have also included an in-depth discussion of the differences in the therapeutic relationship according to the age of the client (adolescent vs. young adult).  *

# Reviewer report: Reviewer response for version 1

**DOI:** 10.5256/f1000research.167098.r322577

**Published:** 2024-10-30

**Authors:** Anna Sidis

**Affiliations:** 1 School of Psychology, University of Wollongong, New South Wales, Australia

This is an interesting study which brings forward the lived experience of adolescents and young people in therapy. Overall the work is clear and well presented, some areas for consideration are:

 1) Given this is qual research, what is the theoretical framework you are applying to the research design and analysis? This should be briefly described in the method section.

 2) The interviews are very short, this must be discussed as a limiting factor as typically qualitative interviews are 60-90 minutes in length. 

 3) The quotes included are very long, it may save space to really look closely at these and try to capture the essence within one or two sentences. 

 4) You have integrated your findings nicely with prior research, I wondered if you can say more about what your findings might challenge about previous knowledge, or what new learnings this study brings. How should/could clinical practice change as a result of the findings of this paper?

Is the work clearly and accurately presented and does it cite the current literature?

Yes

If applicable, is the statistical analysis and its interpretation appropriate?

Not applicable

Are all the source data underlying the results available to ensure full reproducibility?

No

Is the study design appropriate and is the work technically sound?

Partly

Are the conclusions drawn adequately supported by the results?

Yes

Are sufficient details of methods and analysis provided to allow replication by others?

Yes

Reviewer Expertise:

Youth Mental Health, qualitative research, lived experience research, psychotherapy process research.

I confirm that I have read this submission and believe that I have an appropriate level of expertise to confirm that it is of an acceptable scientific standard, however I have significant reservations, as outlined above.

# Response

**Published:** 2025-03-09

**Authors:** Luisa Cassera

**Affiliations:** Catholic University of Sacer Heart, Italy

*Dear Reviewer,*

*Thank you for the valuable feedback.* *We appreciate that you fully grasped the essence of the article and the interest it sparked in us during its development. We have incorporated your suggestions into the article to enhance it both methodologically and in terms of accessibility.*

*Below, you will find the corrections made:*

1. *We have better defined the theoretical background by specifying the design and analysis of the research in the methods section.*
2. *We thank you for pointing out another limitation of the research, which we have promptly included in the dedicated section.*
3. *Regarding the quotations, the decision to include more than one sentence stemmed from the desire not to lose the richness of meaning of each category, clarifying the content of each for the reader. Furthermore, considering the online format of the journal, we felt that the overall increased length of the manuscript was not particularly problematic.*
4. *Regarding the results section, in line with the suggestion from the second reviewer, we have expanded the argument, explaining what new findings the study brings and how it could change clinical practice as a result.*
